# The AEGEAN-169 clade of bacterioplankton is synonymous with SAR11 subclade V (HIMB59) and metabolically distinct

**DOI:** 10.1128/msystems.00179-23

**Published:** 2023-05-18

**Authors:** Eric W. Getz, V. Celeste Lanclos, Conner Y. Kojima, Chuankai Cheng, Michael W. Henson, Max Emil Schön, Thijs J. G. Ettema, Brant C. Faircloth, J. Cameron Thrash

**Affiliations:** 1 Department of Biological Sciences, University of Southern California, Los Angeles, California, USA; 2 Department of Geophysical Sciences, University of Chicago, Chicago, Illinois, USA; 3 Department of Cell and Molecular Biology, Science for Life Laboratory, Uppsala University, Uppsala, Sweden; 4 Laboratory of Microbiology, Wageningen University and Research, Wageningen, The Netherlands; 5 Department of Biological Sciences and Museum of Natural Science, Louisiana State University, Baton Rouge, Louisiana, USA; University of Pretoria, Hatfield, Pretoria, South Africa

**Keywords:** comparative genomics, SAR11, AEGEAN-169, HIMB59, bacterioplankton

## Abstract

Bacterioplankton of the SAR11 clade are the most abundant marine microorganisms and consist of numerous subclades spanning order-level divergence (*Pelagibacterales*). The assignment of the earliest diverging subclade V (a.k.a. HIMB59) to the *Pelagibacterales* is highly controversial, with multiple recent phylogenetic studies placing them completely separate from SAR11. Other than through phylogenomics, subclade V has not received detailed examination due to limited genomes from this group. Here, we assessed the ecogenomic characteristics of subclade V to better understand the role of this group in comparison to the *Pelagibacterales*. We used a new isolate genome, recently released single-amplified genomes and metagenome-assembled genomes, and previously established SAR11 genomes to perform a comprehensive comparative genomics analysis. We paired this analysis with the recruitment of metagenomes spanning the open ocean, coastal, and brackish systems. Phylogenomics, average amino acid identity, and 16S rRNA gene phylogeny indicate that SAR11 subclade V is synonymous with the ubiquitous AEGEAN-169 clade and support the contention that this group represents a taxonomic family. AEGEAN-169 shared many bulk genome qualities with SAR11, such as streamlining and low GC content, but genomes were generally larger. AEGEAN-169 had overlapping distributions with SAR11 but was metabolically distinct from SAR11 in its potential to transport and utilize a broader range of sugars as well as in the transport of trace metals and thiamin. Thus, regardless of the ultimate phylogenetic placement of AEGEAN-169, these organisms have distinct metabolic capacities that likely allow them to differentiate their niche from canonical SAR11 taxa.

**IMPORTANCE:**

One goal of marine microbiologists is to uncover the roles various microorganisms are playing in biogeochemical cycles. Success in this endeavor relies on differentiating groups of microbes and circumscribing their relationships. An early-diverging group (subclade V) of the most abundant bacterioplankton, SAR11, has recently been proposed as a separate lineage that does not share a most recent common ancestor. But beyond phylogenetics, little has been done to evaluate how these organisms compare with SAR11. Our work leverages dozens of new genomes to demonstrate the similarities and differences between subclade V and SAR11. In our analysis, we also establish that subclade V is synonymous with a group of bacteria established from 16S rRNA gene sequences, AEGEAN-169. Subclade V/AEGEAN-169 has clear metabolic distinctions from SAR11 and their shared traits point to remarkable convergent evolution if they do not share a most recent common ancestor.

## INTRODUCTION

SAR11 are aerobic chemoorganoheterotrophs that comprise the largest fraction of bacterioplankton in the global ocean (1). Hallmarks of the group include streamlined genomes with high coding densities and few pseudogenes or gene duplications (2 - 4); unique requirements for amino acids, osmolytes, and C1 compounds (1); and a paucity of canonical regulatory suites (4). Five major SAR11 subclades have been classified and defined through ecogenomic observations during the preceding decades using 16S rRNA gene phylogenetic and whole-genome phylogenomic approaches (1, 5 - 7). SAR11 is currently classified as a taxonomic order (*Pelagibacterales*), and the subclades represent genus to family level distinctions. The majority of SAR11 subclades are found in the epipelagic region, with the predominant subclade being Ia (7); however, subclades Ic and IIb can be found within the mesopelagic and bathypelagic (7 - 10). Surface water genomes have an average size of 1.33 Mbp, contrasting with that of deeper water genomes which average 1.49 Mbp (4, 10). The earliest diverging subclade V comprises two groups—Va shares a surface summer distribution with Ia in the Sargasso Sea, whereas Vb has both a surface and sub-euphotic distribution (7).

Although a stable member of SAR11 in rRNA gene phylogenies (7, 11), the inclusion of subclade V within SAR11 has recently been questioned by advanced phylogenomic approaches using new data (12, 13). Initially, some of these results were questionable due to the availability of only a single genome (HIMB59 [3]) representing subclade V. However, reconstruction of subclade V metagenome-assembled genomes (MAGs) provided additional genomic signal, and the use of methods to correct for compositional biases placed HIMB59-type organisms on a separate branch of the Alphaproteobacteria (13, 14). Nevertheless, analyses of the HIMB59 genome indicated numerous similarities with SAR11, including the small size, low GC content, and conservation of similar metabolic pathways (3). Based on the genomic and ecological similarities with SAR11, a deeper investigation of HIMB59-type organisms is warranted to understand their convergence with SAR11.

Early studies with 16S rRNA gene cloning also defined a sister group to SAR11 that was given the name AEGEAN-169 (15). The group has a cosmopolitan distribution, identified in many regions including the Xiamen Sea, the San Pedro Ocean Time Series (SPOT), the South Pacific Gyre, and the Adriatic Sea (16 - 20). AEGEAN-169 was especially abundant in surface waters of the South Pacific Gyre and the Sargasso Sea, where numerous single-cell genomes were recently obtained, supporting the hypothesis of an ultraoligotrophic lifestyle (18, 19, 21). However, these organisms also respond to phytoplankton blooms (16), and AEGEAN-169 has been observed at depths of 500 m or below at SPOT (17) and at 400 m in the North East Atlantic (22), as well as in coastal (23, 24) and reef (25) habitats. Seasonal blooms of AEGEAN-169 have been identified in the Mediterranean and Xiamen Seas through catalyzed reporter deposition fluorescence *in situ* hybridization (CARD-FISH) and sequencing methodologies, where their abundance was related to elevated CO_2_ concentrations and temperature increases (19, 26). As a result, AEGEAN-169 may play a key role in expanding and warming oligotrophic conditions, globally. AEGEAN-169 have also been implicated in phosphonate consumption (27), implicating another adaptation for the oligotrophic lifestyle.

While our knowledge of this group has improved, the AEGEAN-169 clade has not been examined thoroughly with comparative genomics, nor has its relationship to the SAR11 clade been formally established using modern phylogenomic techniques. AEGEAN-169 have sometimes been classified as belonging to the *Rhodospirillales* (16, 24, 26) and, more recently, were used as an outgroup to SAR11 within the Alphaproteobacteria (21, 27). Here, we present evidence from 16S rRNA gene phylogenetics and phylogenomics that AEGEAN-169 is a heterotopic synonym with SAR11 subclade V, also known as the HIMB59-type clade after the first isolate from the group (3). We do not attempt to reclassify the phylogeny of these organisms, as the close relationship between subclade V/HIMB59 and SAR11 has been examined in detail with advanced phylogenetic methods and appears to result from compositional artifacts (13, 14). Rather, we performed an extensive comparative genomics analysis using publicly available MAGs and single-amplified genomes (SAGs) from multiple databases (21, 28 - 30). We also include a closed genome from the second reported culture of this group, strain LSUCC0245, previously classified as a close relative to HIMB59 (31), and provide the first physiological data for the clade resulting from this isolate. We aimed to define the taxonomy, distribution, and metabolic potential of AEGEAN-169/SAR11 subclade V/HIMB59 to better characterize its relationship to SAR11 *sensu stricto*.

## MATERIALS AND METHODS

### Genome sequencing and assembly of LSUCC0245

We previously isolated a close relative of HIMB59, strain LSUCC0245 (32). Due to the low densities of LSUCC0245 (mid-10^5^ cells/mL) and an inability of this organism to grow in large volumes, 60 50-mL cultures (Supplemental Information—“245_gDNA_010417.pdf” https://doi.org/10.6084/m9.figshare.22027763) grown in JW2 medium (32) were aggregated to achieve sufficient volumes for DNA sequencing. Samples were harvested via 0.2-µm filtration (polycarbonate; Millipore) in the late log phase. DNA was extracted using the Mobio PowerWater kit (Qiagen) with a 50 mL elution in water, and library preparation and sequencing were performed as described (33). Illumina HiSeq sequencing generated 1,925,078 paired-end, 150-bp reads. Genome assembly was performed as described (33). Briefly, reads were trimmed with Trimmomatic v0.38 (34), assembled with SPAdes v3.10.1 (35), and quality checked using Pilon v1.22 (36) after mapping reads to the assembly using BWA 0.7.17 (37). The assembly resulted in a single, circular contig, which was manually rotated approximately halfway between the original overlapping ends. Pilon was run on both the original contig and the rotated contig and detected no issues. Final coverage was 242×. The genome was annotated at IMG (https://img.jgi.doe.gov/) (38).

### Taxon selection

We used SAR11 genomes collected previously from GTDB (30, 33) and AEGEAN-169 genomes from the IMG database, the GORG-TROPICS SAGs database, the Microbiomics database, and the OceanDNA MAG catalog (21, 28, 29, 39). We initially used the HIMB59 and LSUCC0245 genomes, as well as AEGEAN-169 SAGs from GORG-TROPICS and our SAR11 genome collection as a starting data set, and used FastANI v1.33 (40) with default settings to identify additional SAR11 and AEGEAN-169 genomes from the Microbiomics and the OceanDNA MAG data sets. We dereplicated our initial data set of 814 genomes with dREP v3.4.0 (41) using “*dereplicate*” with default settings to produce a final data set of 438 representatives including AEGEAN-169 and the SAR11 clade (Supplemental Information—“genome_metadata.xlsx” https://doi.org/10.6084/m9.figshare.22027763).

### 16S rRNA gene phylogeny

We used barrnap v0.9 (42) to parse all available 16S rRNA genes from the 438 genomes and combined them with relevant AEGEAN-169 16S rRNA gene clones (15), four rRNA gene clones that had been previously classified as SAR11 subclades Va and Vb (7), and other Alphaproteobacteria as outgroups (Supplemental Information—“16S_phylogeny” https://doi.org/10.6084/m9.figshare.22027763). We aligned the gene sequences with Muscle v3.8.1551 (43) using default settings and constructed the tree using IQ-Tree2 v3.8.1551 (44) using “*-b*” for traditional bootstrapping (*n* = 100) and which selected the GTR+F+I+G4 model. The tree was visualized and formatted using iTOL v5 (45). The genomes for which we obtained 16S rRNA gene sequences are listed in the Supplemental Information—“figS2_materials/lin_list.txt” https://doi.org/10.6084/m9.figshare.22027763.

### 16S rRNA gene identity

To calculate 16S rRNA gene identity, we constructed a BLAST (46) database of the 16S rRNA gene sequences from SAR11 and AEGEAN-169 using makeblastdb v2.9.0 with database “*-type nucl*”. We then ran blastn v2.9.0 with “*-perc_identity 40*” and an *e*-value threshold of 1e-15 using the same 16S rRNA gene sequences to generate all pairwise 16S rRNA gene identities.

### Genome metrics

We calculated genome metrics for all genomes in the final data set with CheckM v1.1.3 lineage_wf (47). We ran “*checkm tree_qa*” followed by “*checkm lineage_set*”. Continuing we ran “*checkm analyze*” followed by “*checkm qa*”. Relevant data including genome size, GC content, coding density, genome contamination, and genome completeness resulted from the check output. Estimated genome size was calculated using CheckM metrics (Supplemental Information—“bin_stats_ext.tsv” https://doi.org/10.6084/m9.figshare.22027763) as follows:


$$S=\frac{\alpha (1-\beta )}{\gamma }$$

where α is the number of actual genome base pairs, β is the predicted contamination, and γ is the estimated completeness, as described previously (48).

### Pangenome construction and metabolic profiling

Pangenomic analyses were completed with Anvi’o v7.1 (49). First, we generated Anvi’o contigs databases using “*anvi-gen-contigs-database*”. We then ran a series of annotations, calling the contigs database. For Pfam (50) annotations, we ran “*anvi-run-pfams*”. For NCBI *C*lusters of *O*rthologous *G*roups (COGs) ( 51), we ran “*anvi-run-ncbi-cogs*”. To import Kyoto Encyclopedia of Genes and Genomes (KEGG) (52) annotations, we exported all amino acid sequences from respective contigs databases applying “*anvi-get-sequences-for-gene-calls*”. Amino acid sequences were input into the Ghostkoala (53) web application at KEGG (https://www.kegg.jp/ghostkoala/). Ghoastkoala output was parsed to match respective contigs databases and prepped using “*KEGG-to-anvio*”. To import KEGG functions, we employed “*anvi-import-functions*”. To generate a genome database from the annotated contigs databases, we used “*anvi-get-genomes-storage*”. Having generated a viable genome database, we then employed “*anvi-pan-genome*” with a minbit setting of 0.5 and mcl-inflation set at 2 to construct a pangenome database. To identify enriched functions by subclade, we affixed subclade metadata to the pangenome database using *“anvi-import-misc-data*”. Following this, we ran “*anvi-get-enriched-functions-per-pan-group*” calling COG_category, COG_function, KeggGhostkoala, and Pfam, respectively (54). A pangenome summary was exported via “*anvi-summarize*” (55, 56). The pangenome summary is available in the Supplemental Information—“a169_pang_gene_clusters_summary.tsv” https://doi.org/10.6084/m9.figshare.22027763.

### Phylogenomics

Genomes from AEGEAN-169 and SAR11 clade members were used for phylogenomics with conserved single-copy protein sequences as described previously (57). Briefly, 70 single-copy orthologs were selected from the Anvi’o pangenomics output and all amino acid sequence sets were aligned and trimmed using Muscle v3.8.1551 and Trimal v1.4.1 with the “-automated1” flag (43, 58). The individual alignments were concatenated using the geneStitcher.py script from the Utensils package (https://github.com/ballesterus/Utensils) (59), resulting in a total of 28,836 alignment positions, and the phylogeny was inferred from the unpartitioned, concatenated alignment (Supplemental Information—“phylogenomic tree” https://doi.org/10.6084/m9.figshare.22027763) using IQ-Tree2 v2.0.6 (44), which selected the best-fitting site rate substitution model (LG+F+R10) and “*-bb*” for ultrafast bootstrapping. The tree was visualized and formatted using iTOL v5 (45), with midpoint rooting.

### Proteorhodopsin phylogenetics

To more accurately classify proteorhodopsin diversity across the different predicted variants, orthologous clusters from the Anvi’o pangenomics workflow that were annotated as rhodopsin proteins were aligned with reference sequences provided by O. Beja (personal communication) using Muscle v3.8.1551, culled with Trimal v1.4.1 with the “-automated1” flag, and the phylogeny was inferred using IQ-Tree2 v2.0.6 (44), which selected the best-fitting site rate substitution model (VT+F+G4), and “*-bb*” for ultrafast bootstrapping. The tree was visualized and formatted using iTOL v5 (45). Proteorhodopsin tuning was assigned as previously described (60). The FASTA file containing all sequences and accession numbers for the reference sequences is available in the Supplemental Information—“proteorhodopisn_tree” https://doi.org/10.6084/m9.figshare.22027763.

### Metagenomic recruitment

Metagenomic samples were compiled from the following data sets: TARA Oceans; BIOGEOTRACES; MALASPINA; the Bermuda Atlantic Time Series (BATS); the Chesapeake, Delaware, and San Francisco Bays; the Hawaiian Ocean Time series (HOT); the Columbia River and Yaquina Bay; the Baltic Sea, Pearl River, Sapelo Island, Southern California Bight; and the northern Gulf of Mexico (61 - 69). We recruited reads from all data sets to the AEGEAN-169 genomes *via* RRAP (70 - 72). Post-recruitment, we assessed subclade distribution by summing all *R*eads *P*er *K*ilobases of genome per *M*illion bases of metagenome sequence (RPKM) values for the genomes within each subclade and plotting them by depth, temperature, and salinity.

### Station ALOHA analysis

To assess seasonal distributions of AEGEAN-169, we used data from the HOT data set that contained monthly samples for several different years. We sorted our global recruitment data to parse HOT-specific samples from Station ALOHA for the years 2004–2016. We then summed RPKM values respective to each subclade. We used Ocean Data View to sort summed RPKM data by subclade, month, and depth to interpret seasonality over a 12-month timeline (73).

### Growth experiments

LSUCC0245 was experimentally tested for growth ranges and optima as described previously (32). Briefly, we created artificial seawater media of different salinities through proportional dilution of the major salts. For the temperature-specific experiments, we used the isolation medium, JW2. Growth was measured with flow cytometry as described (32, 74), and growth rates were calculated with sparse-growth-curve (75).

## RESULTS

### Genome reconstruction of LSUCC0245

Strain LSUCC0245 was isolated as previously reported from surface water near the Calcasieu Ship Channel jetties in Cameron, Louisiana, and found to be most similar to HIMB59 based on 16S rRNA gene sequence similarity (31). The two genomes share 99.93% 16S rRNA gene identity. We recovered a complete, circularized genome for strain LSUCC0245 that was 1,493,989 bp with a 32.54% GC content and 1,585 predicted coding genes.

### Phylogenetics and taxonomy

We constructed a 16S rRNA gene tree using all recovered genes from the MAGs, SAGs, and isolates, as well as clones from the original AEGEAN-169 sequence report (15), using SAR11 and other Alphaproteobacteria as outgroups. We also included the subclade Va and Vb sequences previously used to delineate subclade V in SAR11 (7). We found that the Va and Vb sequences corresponded to two monophyletic groups containing all the 16S rRNA gene sequences from our genomes (including HIMB59 and LSUCC0245), as well as the AEGEAN-169 clone library sequences (Fig. S1). This topology demonstrates that the previously designated SAR11 subclade V is synonymous with AEGEAN-169, and we refer to the group by the latter name hereafter. AEGEAN-169 subclade I showed slightly deeper vertical branching in comparison to AEGEAN-169 subclade II.

The average 16S rRNA gene identity between AEGEAN-169 and SAR11 was 82.5% (median: 82.8%, min/max: 80.4%/88%) (Fig. S2 and Supplemental Information—“2_materials/matrix.tsv” https://doi.org/10.6084/m9.figshare.22027763). This indicates a likely family-level difference between AEGEAN-169 and SAR11 but is near the boundary specification for order classification at 82% (76). Also, there were instances of anomalously high identities with SAR11 SAGs, for example, the maximum value between AEGEAN-169 and SAR11 (88%) occurred between the original AEGEAN-169 clone library sequence and a SAR11 SAG (AG-422-B19) that had higher than average identities (~85%) with most of the other AEGEAN-169 sequences. Conversely, the AEGEAN-169 clone library sequence had most identity values near the average for SAR11 versus AEGEAN-169. This may indicate a contaminating 16S rRNA gene sequence in that particular SAG. AEGEAN-169 within subclade I and II gene identities averaged 99.5% (min/max: 97.8%/99.9%) and 98.4% (min/max: 94.7%/99.9%), indicating that subclade I represented a single species, and subclade II represented more than one species. Thus, the AEGEAN-169 clade is at least a distinct family comprising multiple species.

**Fig 1:**
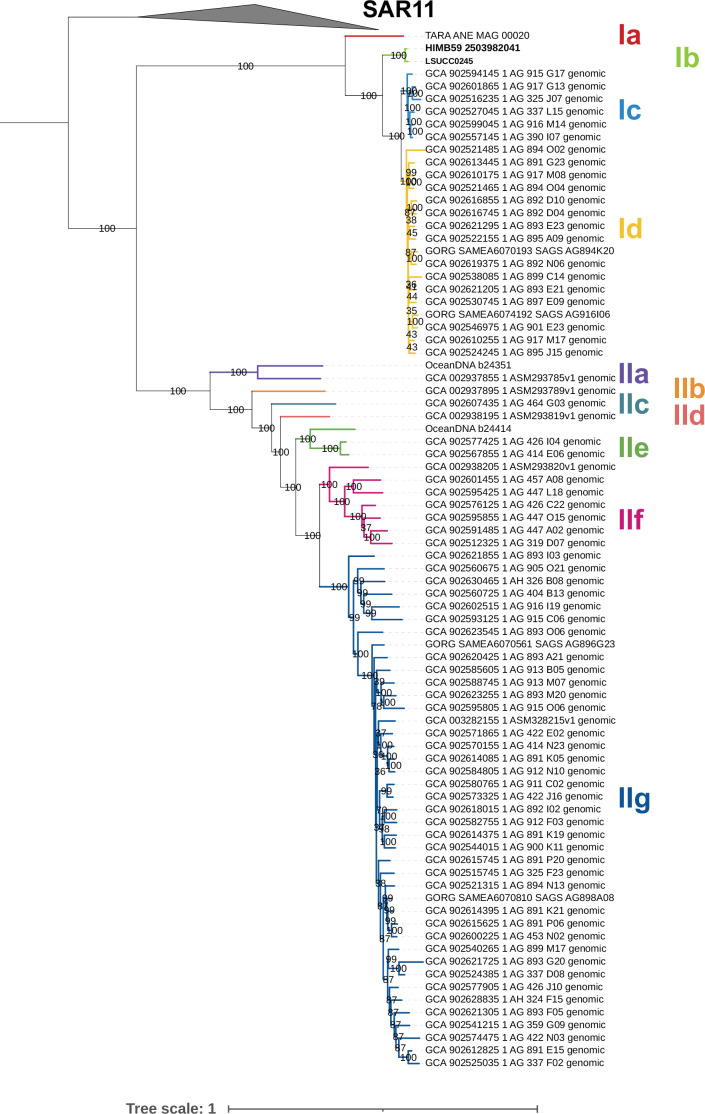
Phylogenomic tree of AEGEAN-169 showing subgroup designations. The tree used a concatenation of 70 single-copy protein sequences with a final alignment of 28,836 amino acid positions. Values on the branches indicate ultrafast bootstrap support (*n* = 1,000), and subclade branches are colored to help provide contrast. Tree scale indicates changes per position according to the scale bar. SAR11 genomes were used as the outgroup.

To investigate the branching pattern between AEGEAN-169 subclades I and II, as well as within each subclade, we also constructed a phylogenomic tree of AEGEAN-169 and SAR11 using orthologous protein sequences extracted from the 438 genomes. The final translated alignment contained 28,837 amino acid positions. The monophyletic grouping of SAR11 and AEGEAN-169 can arise from compositional artifacts (13, 14), and we made no attempt to correct these artifacts here. Rather, we only used SAR11 as an outgroup based on rRNA gene relationships (7, 11) (Fig. S1). Similarly to the 16S rRNA gene tree, we observed two distinct subclades encompassing all AEGEAN-169 genomes wherein strain LSUCC0245 was sister to HIMB059 (Fig. 1). AEGEAN-169 subclade I was characterized by four distinct subgroups (Ia–Id), and subclade II was characterized by seven subgroups (IIa–IIg) defined through branching patterns. LSUCC0245 and HIMB59 were members of subgroup Ib.

### Genome metrics

**Fig 2:**
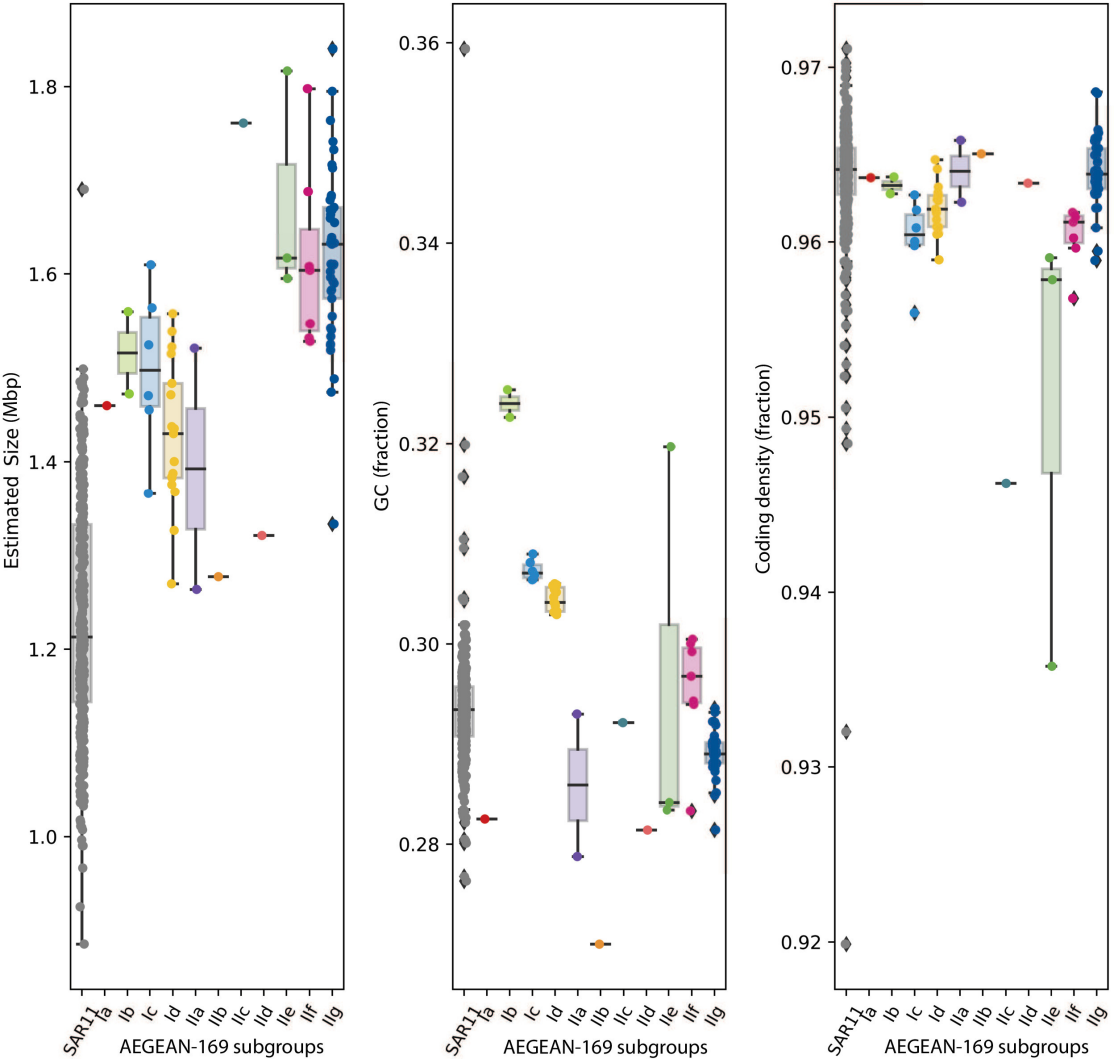
Boxplots illustrating the bulk genome characteristics of AEGEAN-169 subgroups compared to SAR11. Subgroups are colored according to the tree in Fig. 1 and denoted on the *x*-axis. Boxes describe the interquartile range (IQR) with the median indicated as a bar. Whiskers indicate 1.5x IQR, and outlier points are plotted beyond the whiskers. The underlying data points are also plotted on top of each boxplot.

Estimated and actual genome sizes for AEGEAN-169 ranged from 1.26 to 1.84 Mbp with a mean of 1.55 Mbp (Fig. 2). The AEGEAN-169 genomes were larger than SAR11 (*t*-test, *P* << 0.01; R v4.2.1 [77]), which have genomes ranging from 0.88 to 1.69 Mbp, with a mean of 1.22 Mbp). GC content for AEGEAN-169 ranged from 27.0% to 32.5% with a mean of 29.5%. These values were similar to SAR11 (*t*-test, *P* = 0.09), whose GC content ranged from 27.6% to 35.9% with a mean of 29.3%. AEGEAN-169 coding densities ranged from 93.6% to 96.8% with a mean of 96.2%. SAR11 coding densities ranged from 92.0% to 97.1% with a mean of 96.4%. Thus, AEGEAN-169 had similar levels of genome streamlining to SAR11 even though the genomes were slightly larger.

### Ecology

AEGEAN-169 was predominantly a surface water organism within the euphotic zone, with subgroup IIg dominating metagenomic recruitment in most marine locations, followed by subgroup Id (Fig. S3). Subgroup IIc appeared to be a deep water bathytype, recruiting reads almost exclusively below 125 m, with highest recruitment below the euphotic zone. Subgroup IIe was also more abundant in deeper waters, although it could be found at the surface (Fig. S3). These patterns were consistent with distributions by temperature, where the surface subclades dominated in warmer temperatures, and the deeper subclades recruited most reads in colder water (Fig. S4). We classified salinity according to the Venice system (<0.5 fresh, 0.5–4.9 oligohaline, 5–17.9 mesohaline, 18–29.9 polyhaline, 30–39.9 euhaline, >40 hyperhaline) (ITO 1959 [78]), confirming subgroup IIg as marine organisms with recruitment almost exclusively in euhaline and hyperhaline water (Fig. S5). Subgroup Ib was most prominent in polyhaline samples and recruited the most reads from mesohaline samples, so this likely represents a brackish water clade. None of the genomes within any subgroup represented freshwater taxa.

**Fig 3:**
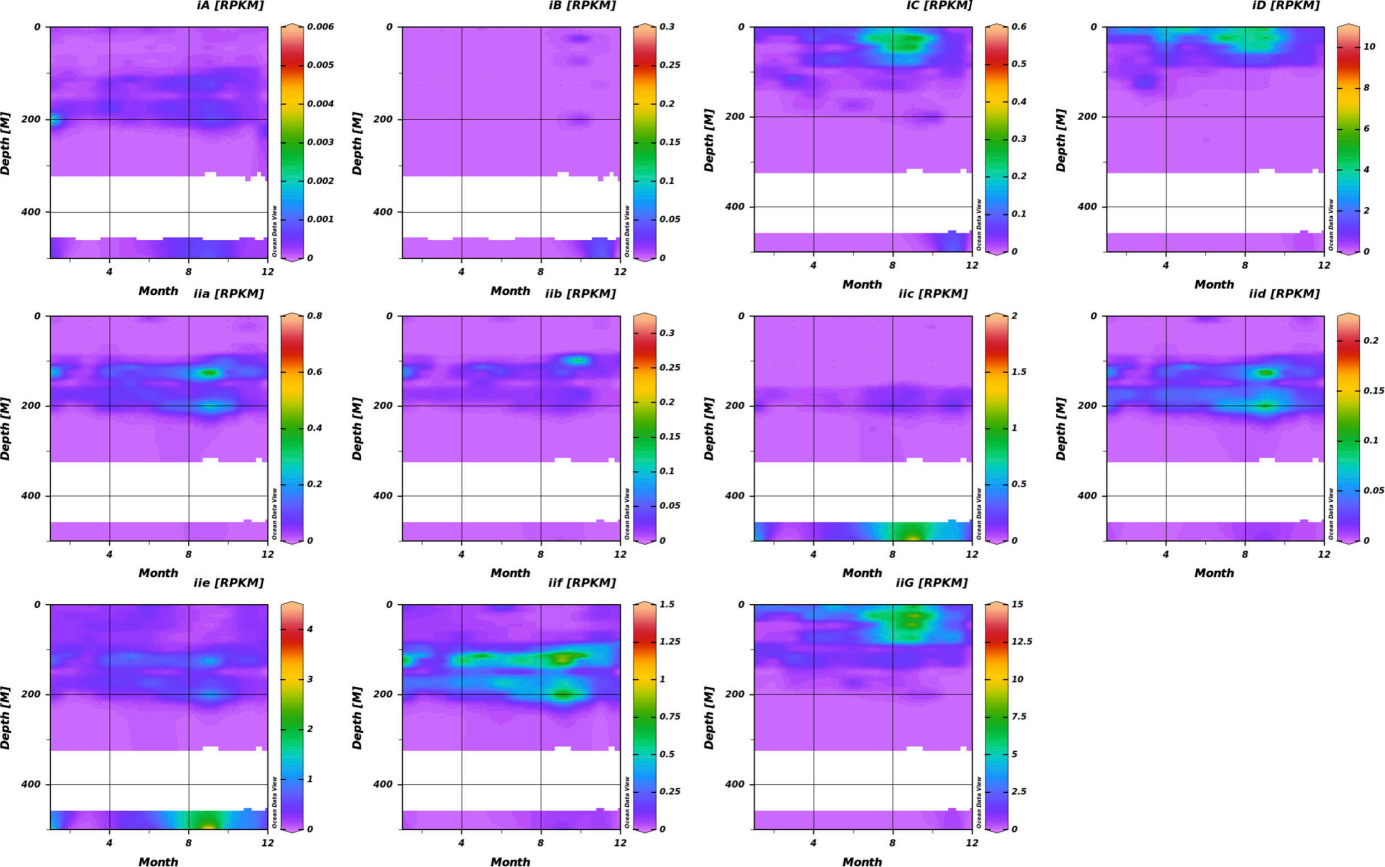
AEGEAN-169 subgroup distribution at Station ALOHA using HOT data spanning from 2003 to 2016. Each subgroup is plotted with a separate scale. Months correspond from 1 (January) to 12 (December). RPKM, Reads Per Kilobase (of genome) per Megabase (of metagenome).

We also examined spatiotemporal trends from the HOT using samples collected at Station ALOHA monthly during the years 2003–2016 and normalizing by month. These samples extended to 500 m. The data indicated that AEGEAN-169 has two primary ecological niches at Station ALOHA; surface water subgroups that bloom in the late summer/early fall and subgroups that occur primarily at 100–200 m and appear to have a fall bloom period (Fig. 3). Subgroups Ic, Id, and IIg were the primary surface water groups, with Id and IIg being the most abundant at Station ALOHA, consistent with our global recruitment data (Fig. S3 to S5). Surface water temperatures at Station ALOHA have ranged from approximately 24°C–26°C over a 30-year timespan (79), and we found subgroups Id and IIg predominantly in temperatures above 20°C (Fig. S4). Subgroups IIa, IId, and IIf were the dominant ecotypes in the 100–200 m range, suggesting they are associated with the deep chlorophyll maxima. Subgroups IIc and IIe were the only clades detected at 500 m, consistent with these organisms being deep water bathytypes.

### Metabolic variation

**Fig 4:**
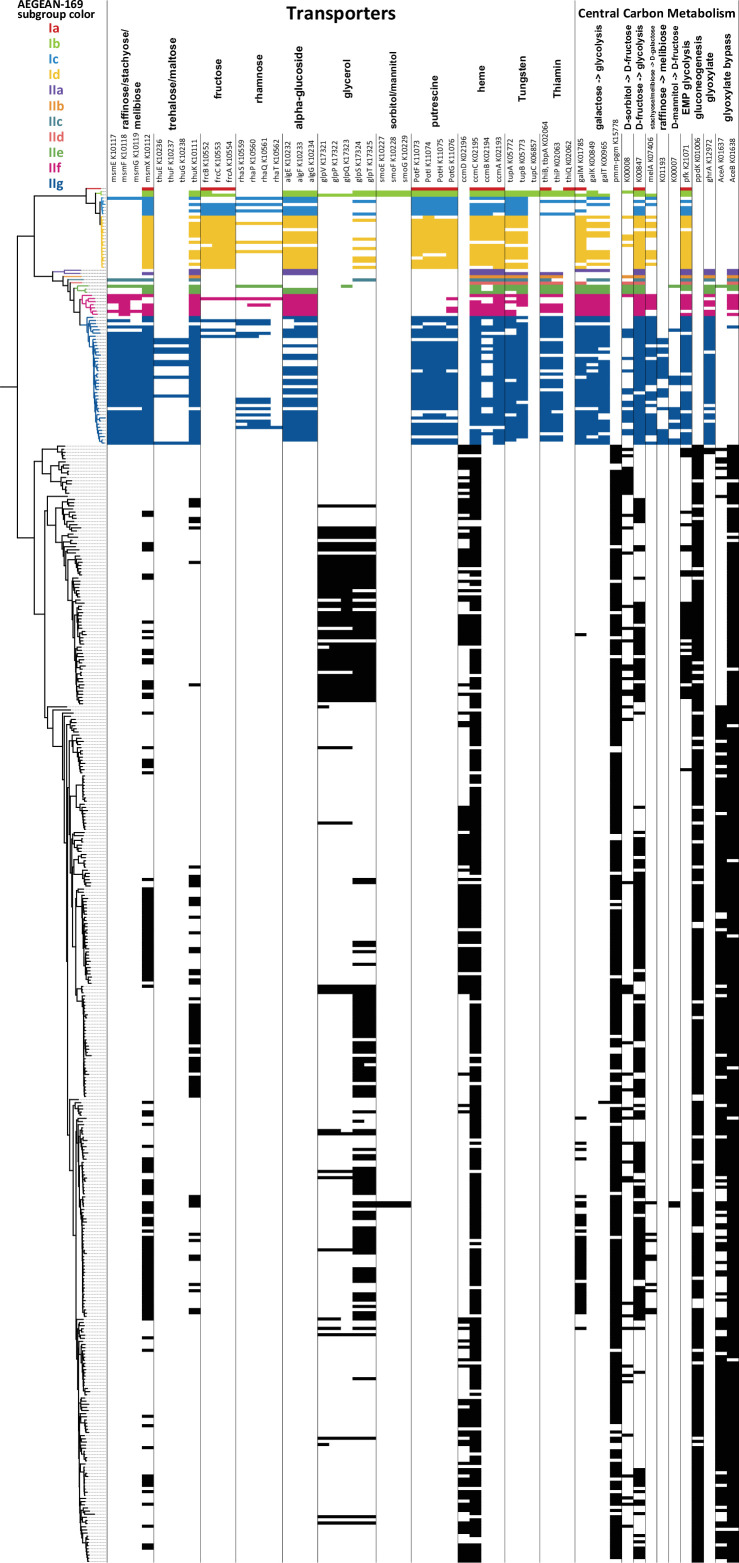
Key metabolic variation between AEGEAN-169 and SAR11. The phylogenomic tree on the left has colors by AEGEAN-169 subgroup according to the key, with SAR11 indicated in only black branches below AEGEAN-169. Gene names correspond to components found for these systems.

What is currently known about the metabolism of AEGEAN-169 comes primarily from the HIMB59 genome (3). We have extended these observations to a larger diversity of genomes spanning the two subclades of AEGEAN-169. In general, these organisms were predicted to be obligate aerobes with chemoorganoheterotrophic metabolism. They had genes for central carbon metabolism by way of glycolysis, the pentose phosphate pathway, and the citric acid cycle, similar to SAR11. However, AEGEAN-169 metabolic capacity differed in several important ways, notably through sugar metabolism and trace metal and vitamin transport. AEGEAN-169 genomes had a fructose ABC transporter, predominantly in subclade I, subclade II members had a predicted trehalose/maltose ABC transporter, and both subclades included representatives with a galactose/raffinose/stachyose/melibiose ABC transporter systems that were not found in SAR11 (Fig. 4). Although AEGEAN-169 genomes lacked an L-proline symporter found in SAR11, they shared the *potABCD* putrescine/spermidine transporter with SAR11 and had an additional *potFGBI* putrescine transporter and *algEFG* alpha-glucoside transporter not found in SAR11 (Fig. 4). Moreover, both AEGEAN-169 subclades had greater transport potential for trace metals and vitamins. Both subclades had heme and tungsten transporters not found in SAR11, as well as the potential for thiamin transport that was absent in SAR11 (Fig. 4).

AEGEAN-169 glycolytic inputs and central carbon metabolism also had key differences from those in SAR11. As reported previously for HIMB59 (3), AEGEAN-169 had the phosphofructokinase (pfk) for Embden–Meyerhof–Parnas glycolysis (Fig. 4). While this gene was found in some SAR11, including LD12 (80), it was missing from the dominant SAR11 subclade Ia organisms (Fig. 4). Consistent with the transporters for sugars, sugar metabolism was expanded. AEGEAN-169 members had predicted genes for the conversion of many sugars into galactose and/or fructose, as well as the *galKMT* pathway for galactose metabolism (Fig. 4). AEGEAN-169 also differed from SAR11 through the absence of *ppdK*, which converts phosphoenolpyruvate to pyruvate for gluconeogenesis (Fig. 4). While some subclade II members had *aceB* (malate synthase), we only found two examples of *aceA* (isocitrate lyase) in AEGEAN-169, and thus they appear to mostly lack the traditional glyoxylate shunt that is a hallmark of SAR11 (3, 80). However, most members of AEGEAN-169 subclade II had a predicted *ghrA* (glyoxylate/hydroxypyruvate reductase) (Fig. 4)*,* which can convert glycolate to glyoxylate. Only two LD12 genomes had this gene within SAR11. AEGEAN-169 organisms with both *ghrA* and *aceB* should have the ability to bring glycolate into the TCA cycle, allowing them to take advantage of that widely abundant phytoplankton-produced compound (81, 82).

### Proteorhodopsin

We identified multiple gene clusters annotated as potential rhodopsin homologs within the pangenome, and numerous AEGEAN-169 genomes, including LSUCC0245, contained multiple copies of predicted proteorhodopsins (Fig. S6). Sometime these copies were quite divergent. For example, LSUCC0245 had one proteorhodopsin copy in each of the arbitrary AEGEAN-169 proteorhodopsin clades I and V, and these two copies were predicted to have different spectral tuning: one blue and one green. This pattern of differential tuning in proteorhodopsin duplicates was seen in other AEGEAN-169 organisms as well (Fig. S6). In addition, we found a separate group of possible rhodopsin homologs that currently do not have functional prediction. Thus, AEGEAN-169 has a wide diversity of proteorhodopsin sequences and numerous instances of phylogenetically and spectrally divergent copies within individual genomes. These observations corroborate a recent investigation of proteorhodopsin paralogs in SAR11 and HIMB59-clade organisms (83).

### Physiology

**Fig 5:**
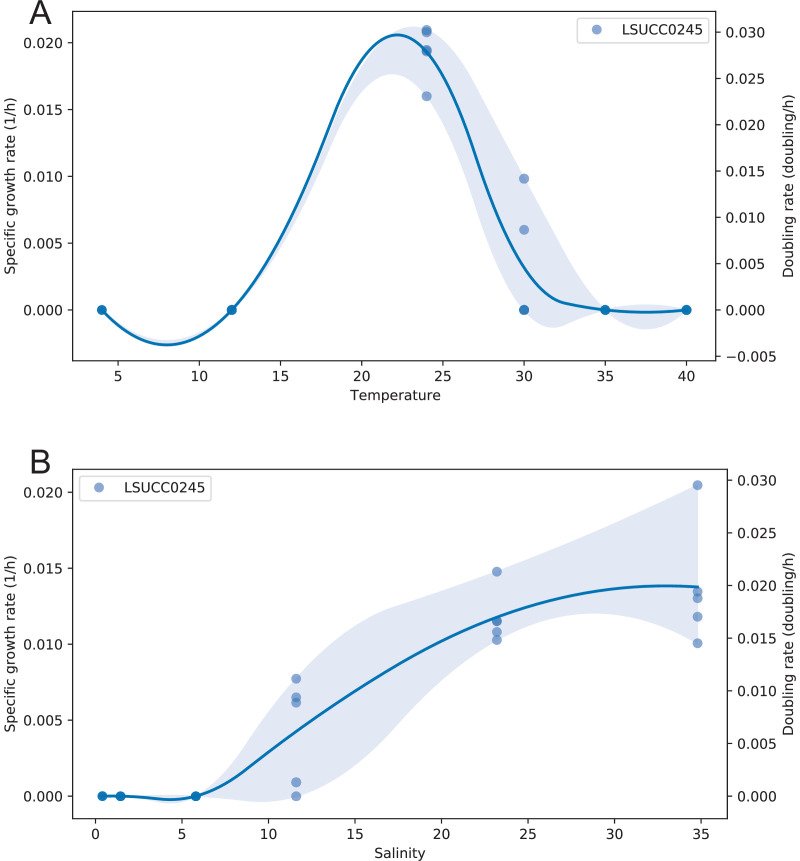
LSUCC0245 temperature-dependent (**A**) and salinity-dependent (**B**) growth. Calculated using sparse-growth-curve (75). Specific growth rates and doubling rates are indicated with the dual *y*-axes. An interpolation connects the points to predict rates in between measured values, and shading indicates 95% confidence intervals.

We measured the growth rates of LSUCC0245 across multiple salinities and temperatures. This strain was a marine-adapted mesophile, growing optimally at 24°C, and slowly at 30°C, but not at 12°C or 35°C (Fig. 5A). It grew optimally at a seawater salinity of 34 and in salinities as low as 11.6. Its maximum growth rate was 0.02 ± 0.007 divisions per hour at 24°C (Fig. 5B). LSUCC0245 had very low growth yields in our media (<10^6^ cells/mL) (Fig. S7). Given the complex mixture of low concentration carbon sources in the medium, it appears likely that LSUCC0245 was only using a small subset of the available substrates. We also note that several of the sugar, sugar alcohol, and polyamine compounds that we predict as usable by LSUCC0245 (e.g., sorbitol, mannitol, fructose, galactose, putrescine; Fig. 4) were not available in the JW2 medium (32). Thus, more in-depth exploration of usable carbon sources is warranted.

## DISCUSSION

This study aimed to define AEGEAN-169 through the lens of taxonomy, ecology, and metabolism, with the goal of understanding how similar or distinct these organisms are from SAR11. Our results provide the first detailed examination of AEGEAN-169 genomics and genome-based ecology. The overall picture is one of a group that shares a very similar ecological regime as SAR11—the majority of AEGEAN-169 members are most abundant in surface marine waters with seasonality that overlaps with SAR11. AEGEAN-169 and SAR11 were similar in relation to central carbon metabolism with a few key differences in capability. However, there were important metabolic differences between these groups, particularly the utilization and transport of additional sugars, trace metals, and vitamins by AEGEAN-169 that may help distinguish their niche in terms of interactions with dissolved organic matter.

Although previous phylogenetic studies have considered SAR11 and AEGEAN-169 sister clades (21, 27, 84), this relationship likely results from compositional artifacts in the underlying sequence data (12, 13, 15). We reemphasize that our goal with phylogenetics and phylogenomics in this study was only to establish the subclade relationships within AEGEAN-169. Our work demonstrates, using both 16S rRNA genes and whole-genome data, that AEGEAN-169 is a heterotopic synonym with SAR11 subclade V/HIMB59 (Fig. S1), thus condensing these disparate taxonomic designations. Given its historical precedent, we propose using AEGEAN-169 as the primary moniker as we have done herein until a formal taxonomic designation is established. The major AEGEAN-169 subclade I and II delineations corresponded to the Va and Vb designations made on the basis of previous 16S rRNA gene phylogeny, respectively (7). While early work suggested a closer taxonomic relationship between HIMB59 and SAR11 through the use of synteny and genome organization (3), these observations were based on a singular genomic representative from AEGEAN-169 subclade I (HIMB59) and did not define the depth of the genus, as is now possible with current data sets (21, 28, 29). Thus, future examination of the phylogenetic relationships between AEGEAN-169, SAR11, and other Alphaproteobacteria should benefit from the expanded taxon selection provided by these studies.

Members of AEGEAN-169 were primarily surface water marine organisms, sharing similar ecological distributions with SAR11 (7, 85 - 87). AEGEAN-169 was most abundant at depths between 5.1 and 75 m. These observations corroborate previous characterizations of AEGEAN-169 as a predominantly surface water group that is likely stimulated by blooms occurring in late summer and fall (16, 18, 19, 88). AEGEAN-169 subgroups IIc and IIe were found predominantly in deeper waters (Fig. S3) supporting bathytype designations, similarly to SAR11 subclade Ic (10). Notably, subclade II was the primary group with recruitment observed at 200 m or below, which is consistent with its distribution based on 16S rRNA gene data at BATS where subclade I (Va) was a surface water group, whereas subclade II (Vb) was found in both surface and 200 m waters (7). With respect to salinity, AEGEAN-169 were almost all marine-adapted, although subgroup Ib was most abundant in polyhaline conditions (Fig. S5) (12). Subgroup Ib contains only the two isolates. The fact that the cultured representatives (LSUCC0245 and HIMB59) branched together and separately from the rest of the AEGEAN-169 subclades despite being isolated from the Gulf of Mexico and the North Pacific gyre at first seems unlikely. However, most of the available AEGEAN-169 genomes were collected from BATS (21), in the North Atlantic open ocean, whereas the two cultures were isolated from samples collected in coastal locations (3,32). We also observed that LSUCC0245 was capable of growing in brackish salinities common in coastal systems (Fig.5B). Thus, the combination of metagenomic recruitment data, growth physiology, and isolation locations suggests that subclade Ib represents a group of coastal specialists within AEGEAN-169. The specific subgroup salinity preference resembles that described for SAR11 subclade IIIa (33). Overall, the high amount of overlap between the habitats of SAR11 and AEGEAN-169 likely explains the metabolic variation we observed between the two groups.

AEGEAN-169 lacked *ppdK* which converts phosphoenolpyruvate to pyruvate as well as the converse reaction (Fig. 4). This suggests that gluconeogenic activity is limited, which differs from the predicted complete gluconeogenesis pathway in SAR11 (3). Novel sugar intake was exhibited in AEGEAN-169 by means of multi-alpha-glucoside, fructose, rhamnose, trehalose/maltose, and raffinose/stachyose/melibiose ABC transporters (Fig. 4). Expanded sugar metabolism was a feature first reported for HIMB59 based on the single genome at the time (3), and we demonstrate that this trait is conserved across AEGEAN-169 genomes. Many of the aforementioned sugars were predicted to be metabolized to galactose and through the *galKMT* genes (missing in SAR11) to alpha-D-glucose-1P (Fig. 4). However, AEGEAN-169 was missing the phosphoglucomutase found in SAR11, and we found no other means to convert alpha-D-glucose-1P to alpha-D-glucose-6P. Thus, how these sugars enter glycolysis is currently unclear. Nevertheless, given the greater emphasis on sugar transport and metabolism, but the lack of *ppdK*, perhaps AEGEAN-169 organisms rely on external sources of sugar in place of gluconeogenesis.

Another difference was that most AEGEAN-169 members had a predicted putrescine ABC transporter (*potFGHI*) not found in SAR11 (Fig. 4). SAR11 and some AEGEAN-169 members have homologs of the *potABCD* spermidine/putrescine ABC transporter (Supplemental Information—“a169_pang_gene_clusters_summary.tsv” https://doi.org/10.6084/m9.figshare.22027763), and SAR11 responds disproportionately to the addition of both of these polyamines in natural communities (89, 90). The *potABCD* genes transport five different polyamines in SAR11, where these compounds can meet cellular nitrogen requirements (91) and is spermidine-preferential in *Escherichia coli* (92). The additional *potFGHI* genes in AEGEAN-169 suggest increased use of putrescine compared to SAR11, as this transporter is considered putrescine-specific (92). Thus, SAR11 and AEGEAN-169 may have differential polyamine preferences in nature.

Trace metal and vitamin transport also distinguished AEGEAN-169 from SAR11. AEGEAN-169 uniquely had genes for an iron/zinc chelator, as well as heme and tungsten transport (Fig. 4). SAR11 members have quite limited trace metal transport capabilities (93). The potential of AEGEAN-169 to transport heme would provide them with an alternative source of iron, and the presence of the transporter corroborates recent findings that many abundant marine microorganisms are heme auxotrophs (94), including AEGEAN-169 members (designated HIMB59 by the authors). Most surprising was the presence of a tungsten transporter which traditionally has been observed in thermophilic archaea as well as *Sulfitobacter dubius* and some *Clostridium* spp. and *Eubacterium* spp., although hyperthermophilic archaea appear to be the only group that requires tungsten (95 - 97). This suggests that AEGEAN-169 may utilize tungstoenzymes, such as a tungsten-containing version of formate dehydrogenase (98). Formate dehydrogenases are conserved throughout SAR11 and AEGEAN-169 (Supplemental Information—“a169_pang_gene_clusters_summary.tsv” https://doi.org/10.6084/m9.figshare.22027763), but the clades may use different cofactors. AEGEAN-169 also had the capacity to transport thiamin (vitamin B1), which may provide another means of niche differentiation since SAR11 relies on thiamin precursors instead of directly uptaking thiamin (99).

The increased potential of sugar, trace metal, and vitamin transport and metabolism are important traits differentiating AEGEAN-169 from SAR11, and likely mean that AEGEAN-169 has a more extensive metabolic niche than SAR11. This expanded metabolic repertoire correlates with the slightly larger genome sizes in AEGEAN-169 compared to SAR11, even though both strains have the hallmark coding density associated with genome streamlining. Nevertheless, SAR11 is the more successful group, with relative abundances that are usually much higher than that of AEGEAN-169 (e.g., Ref. [7]). In this context, it is notable that strain LSUCC0245 grew to much lower cell densities than SAR11 strains in the same medium, even though growth rates were similar (Fig. S7) (33, 80). Since our defined media have numerous carbon compounds at similar concentrations, these yield differences either mean that SAR11 and AEGEAN-169 use a different set of compounds available in the medium, or there is something inherently different about growth physiology with AEGEAN-169. Future studies should incorporate cultivation assessments to investigate the metabolic differences we have identified, as well as the differences in physiology. Additional isolates will also help improve our overall understanding of the diversity of functions in the group and shed more light on the evolutionary pressures that have led to the similarities that AEGEAN-169 and SAR11 share.

## Data Availability

Raw reads for the LSUCC0245 genome were deposited at NCBI BioProject no. PRJNA931292, and the genome is publicly available on IMG (https://img.jgi.doe.gov/) under Genome ID: 2756170191. Supporting data sets, scripts, and files, including those for generating figures, are available at https://doi.org/10.6084/m9.figshare.22027763.

## SUPPLEMENTAL MATERIAL

FIG S1 msystems.00179-23-s0001.eps 16S rRNA gene tree phylogeny of AEGEAN-169, SAR11, and other Alphaproteobacteria. Genomes and clone library markers of interest are bolded within the AEGEAN-169 subclades for emphasis. All sequences for SAR11 are collapsed. The original AEGEAN-169 16S rRNA gene marker groups with the SAR11 subclade Va markers and the two cultured isolates, HIMB59 and LSUCC0245 in subclade I. The SAR11 subclade Vb markers group with AEGEAN-169 subclade II. Values at the nodes indicate traditional bootstraps (n=100) and the Tree scale indicates changes per position according to the bar. The tree was rooted on *Rickettsia typhi*.

FIG S2 msystems.00179-23-s0002.eps 16S rRNA gene identity of AEGEAN-169 and SAR11**.** Percent identity is denoted according to the scale bar on the right.

FIG S3 msystems.00179-23-s0003.eps AEGEAN-169 subgroup distribution by depth**.** Subgroups are plotted according to the sum of the individual genome RPKMs comprising that subgroup. RPKM - Reads per kilobase of genome sequence per megabase of metagenomic sequence.

FIG S4 msystems.00179-23-s0004.eps AEGEAN-169 subgroup distribution by temperature**.** Subgroups are plotted according to the sum of the individual genome RPKMs comprising that subgroup. RPKM - Reads per kilobase of genome sequence per megabase of metagenomic sequence.

FIG S5 msystems.00179-23-s0005.eps AEGEAN-169 subgroup distribution by salinity**.** Subgroups are plotted according to the sum of the individual genome RPKMs comprising that subgroup. RPKM - Reads per kilobase of genome sequence per megabase of metagenomic sequence. Salinity categories are as follows: < 0.5 fresh, 0.5-4.9 oligohaline, 5-17.9 mesohaline, 18-29.9 polyhaline, 30-39.9 euhaline, > 40 hyperhaline.

FIG S6 msystems.00179-23-s0006.eps Proteorhodopsin phylogeny. Genomes with multiple copies of proteorhodopsin have a 1, 2, or 3 following the node labels at the tips. Ultrafast bootstrap values (n=1000) are indicated on the branches and only clades with AEGEAN-169 members are highlighted. Blue and green highlighting corresponds to the predicted spectral tuning of those groups. Clade names are arbitrary to distinguish AEGEAN-169 proteorhodopsin spectral and phylogenetic diversity. The red highlight for Clade VI indicates an undetermined function. Tree scale indicates changes per position according to the scale bar.

FIG S7 msystems.00179-23-s0007.eps Growth data for LSUCC0245 temperature and salinity experiments. These data underlie the computed rates in Figure 5.

ASM does not own the copyrights to Supplemental Material that may be linked to, or accessed through, an article. The authors have granted ASM a non-exclusive, world-wide license to publish the Supplemental Material files. Please contact the corresponding author directly for reuse.
